# Metastatic Prostate Cancer Presenting as Fatigue in an Older Adult

**DOI:** 10.1002/ccr3.72164

**Published:** 2026-03-05

**Authors:** Arianna R. Tidball, Elizabeth N. Harlow

**Affiliations:** ^1^ College of Medicine University of Nebraska Medical Center College of Medicine Omaha USA; ^2^ Division of Geriatrics, Gerontology and Palliative Medicine University of Nebraska Medical Center Omaha USA

**Keywords:** aging, fatigue, geriatrics, prostate cancer

## Abstract

Fatigue in older adults, though common and nonspecific, may signal serious disease. This report describes an older man whose complaint of fatigue, evaluated with urinary symptoms, revealed metastatic prostate cancer with diffuse bone involvement. This highlights the need to consider malignancy in the differential of unexplained fatigue.

## 1 Introduction

Fatigue is a common, yet often underappreciated, primary complaint in older adults. It comes with a broad differential diagnosis that spans from benign to life‐threatening conditions. While typically attributed to factors such as chronic disease or medication side effects, fatigue can also be a sign of an underlying malignancy. Prostate is the most common cancer in men and the second leading cause of cancer‐related death in men in the United States [1]. Although early‐stage disease is often asymptomatic and diagnosed through screening, advanced prostate cancer can present subtly, including with constitutional symptoms like fatigue, bone pain, or laboratory abnormalities. This report describes an older adult man whose primary complaint of fatigue, which was evaluated in the context of urinary symptoms, led to the diagnosis of high‐grade metastatic prostate cancer with diffuse bone involvement.

### 1.1 Objectives

This case report aims to:

- Highlight the diagnostic value of a structured workup for fatigue in older adults, particularly when accompanied by nonspecific constitutional symptoms.
- Describe an example of atypical presentation of metastatic prostate cancer, with a focus on non‐painful skeletal involvement and general systemic symptoms.
- Emphasize the clinical relevance of abnormal lab values such as elevated alkaline phosphatase as a potential indicator of bone metastases, particularly in the absence of liver pathology.
- Encourage clinicians to maintain a high index of suspicion for malignancy when evaluating fatigue, especially in patients with genitourinary symptoms or abnormal lab values.

## 2 Case History/Examination

Mr. X is an 81‐year‐old man who presented with significant exhaustion and a recent loss of taste. He had been active until several weeks prior, when he developed significant worsening of baseline fatigue. His past medical history includes hyperlipidemia, hypertension, atrial fibrillation, sleep apnea, anxiety, depression, and osteoarthritis. He also reported increased urinary frequency over the past year, stating that he was only able to sleep for 1 h at a time between trips to the bathroom. He attributed his fatigue to his interrupted sleep cycle. To help manage these symptoms, he was prescribed tamsulosin, finasteride, and oxybutynin. A cystoscopy with transurethral resection of the prostate (TURP) was planned to address his urinary incontinence.

At the time of his visit, the only other major symptom was a 3‐day history of loss of taste. He denied any other upper respiratory or COVID‐19‐related symptoms. A COVID‐19 test was performed and returned negative.

## 3 Investigations/Treatment

Routine labs were ordered, which revealed a markedly elevated alkaline phosphatase level (562 U/L; normal adult range 44 to 147 U/L), while all other results were within normal limits. A gamma‐glutamyl transferase (GGT) test was subsequently ordered to help identify the source of the elevated alkaline phosphatase and was normal (27 U/L; normal adult range 5 to 40 U/L), suggesting a bony origin. A nuclear medicine bone scan was then performed, revealing widespread abnormal radiotracer uptake throughout the axial skeleton and portions of the appendicular skeleton. These findings were highly suggestive of widespread osseous metastases. A Prostate‐Specific Antigen (PSA) test was subsequently ordered and showed a markedly elevated PSA level (1421.09 ng/mL; normal adult range 0 to 4.0 ng/mL). A prostate biopsy performed in mid‐May confirmed prostatic adenocarcinoma with a Gleason score of 9/10 with 50% tumor involvement in more than 10 sampled cores. Following his TURP procedure, Mr. X's PSA level decreased from 1421.09 ng/mL to 271.75 ng/mL. He was subsequently referred to oncology for further management. With initiation and completion of his treatment, his PSA levels continued to decline to 4.34 ng/mL and ultimately to < 0.05 ng/mL over the course of 6 months.

## 4 Discussion

### 4.1 Working up a General Complaint of Fatigue

Fatigue is a common yet non‐specific clinical complaint, accounting for approximately 5%–10% of U.S. primary care visits [2] and up to 10%–20% of visits worldwide [3]. It can be a manifestation of a range of conditions and requires a structured approach to identify treatable or potentially life‐threatening causes, beginning with a thorough history and physical examination. Key pieces of the patient's history include onset, duration, constitutional symptoms such as weight loss, night sweats, or pain, and changes in daily functioning [3]. A detailed review of systems can also help localize the origin of fatigue and differentiate between physiological, psychological, metabolic, or chronic disease causes [2].

In Mr. X's case, his age, polypharmacy, and known cardiovascular and psychiatric comorbidities made the initial differential diagnosis very broad. His chief complaint of severe fatigue, coupled with vague systemic symptoms such as urinary frequency and a recent loss of taste, prompted a guideline‐based laboratory evaluation for fatigue, including a complete blood count (CBC), basic metabolic panel (BMP), thyroid‐stimulating hormone (TSH), erythrocyte sedimentation rate (ESR), C‐reactive protein (CRP), and liver function tests (LFTs) [4].

One of the most notable abnormalities was a markedly elevated alkaline phosphatase, which ultimately guided the diagnostic process. While alkaline phosphatase is a non‐specific enzyme that can indicate hepatobiliary or bone pathology [5], the presence of a normal GGT helped localize the source to the skeletal system rather than the liver [6]. This differentiation prompted further investigation with a nuclear medicine bone scan, a guideline‐supported tool for detecting osseous metastases in patients with biochemical or clinical suspicion of malignancy [7]. In Mr. X's case, this stepwise approach facilitated the process of going from a vague biochemical finding to a diagnosis of diffuse metastatic disease, explaining his progressive fatigue and systemic decline. This progression from routine lab work to diagnostic imaging underscores the diagnostic value of targeted testing, particularly in older adults where serious conditions may present subtly.

### 4.2 Prostate Cancer and Bone Metastasis Presentation

Prostate cancer is the most common solid malignancy in men and often presents silently until it metastasizes, with bone being the most common site of metastasis [8]. Fatigue in this context may result from systemic effects of malignancy, cytokine‐mediated inflammation, or bone marrow involvement. In older adults, particularly men with urinary complaints or elevated alkaline phosphatase, metastatic prostate cancer should be a key consideration in the differential. Mr. X's markedly elevated PSA (1421.09 ng/mL) and confirmatory prostate biopsy established the diagnosis of high‐grade metastatic prostatic adenocarcinoma. It is worth noting that PSA alone is often not sufficient to raise concern for prostate cancer unless the value is especially elevated, as it was in Mr. X's case.

A unique aspect of Mr. X's clinical journey was the lack of musculoskeletal pain prior to diagnosis. This raises an important question: what is the clinical significance of the absence of pain in metastatic disease? In prostate cancer, bone metastases may not initially present with persistent or severe pain; lesions can remain clinically silent or produce fluctuating symptoms, particularly if the metastases are sclerotic rather than lytic [9]. Additionally, the subjective experience of pain may be diminished by age‐related changes in pain perception, comorbidities such as osteoarthritis, or chronic and concurrent use of pain‐relieving medications. Bone metastases from prostate cancer frequently led to skeletal‐related events (SREs), including pathological fractures, spinal cord compression, and hypercalcemia, which contribute significantly to morbidities [10]. These potential complications call attention to the significance of detecting bone metastases early, even when pain symptoms are absent.

### 4.3 Detecting Metastatic Prostate Cancer in Non‐Target Populations

This case illustrates the value of a systematic and guideline‐informed approach to fatigue, especially in older adults, among whom fatigue is a common complaint and may mask more serious conditions [11]. Additionally, it highlights the value of interpreting laboratory abnormalities, such as Mr. X's elevated alkaline phosphatase, in the context of clinical symptoms while also utilizing clinical reasoning to uncover possible underlying conditions. Furthermore, this case underscores the importance of maintaining vigilance for symptoms in older adults that may indicate serious underlying conditions.

In Mr. X's case, appropriate screening had been completed; however, his cancer developed after the recommended screening window had closed. Current guidelines from the U.S. Preventive Services Task Force (USPSTF) advise against routine PSA screening for men aged 70 years and older [12]. Notably, men aged 75 years or older accounted for 43.5% of distant metastatic prostate cancer cases in the Surveillance, Epidemiology, and End Results (SEER) database, with incidence rates rising by 6.5% annually between 2011 and 2018 [13]. This increase coincided with the USPSTF's recommendations against PSA‐based screening in older adults, suggesting a potential association between reduced screening and higher rates of late‐stage diagnoses in this population. Mr. X's case illustrates the challenges and nuances of balancing screening guidelines with timely diagnoses and symptom management in the aging population.

## 5 Conclusion

This case emphasizes the importance of a systematic and guideline‐informed approach when evaluating fatigue in older adults. While fatigue is a common and non‐specific symptom, it can occasionally serve as the warning sign of a serious underlying pathology. In Mr. X's case, a workup initiated for fatigue and urinary symptoms uncovered high‐grade metastatic prostate cancer. Remarkably, his disease included diffuse skeletal metastases despite little to no pain. Clinicians should consistently consider malignancy in the differential diagnosis of fatigue in older adults, especially when supported by specific clinical or laboratory findings. Early recognition and appropriate referrals can significantly impact patient outcomes.

## Author Contributions

**Arianna R. Tidball:** writing – original draft. **Elizabeth N. Harlow:** supervision, writing – review and editing.

## Funding

The authors have nothing to report.

## Consent

A written informed consent was obtained from the patient to publish details of his case in this report in accordance with the patient consent policy.

## Data Availability

The data that support the findings of this study are available on request from the corresponding author. The data are not publicly available due to privacy or ethical restrictions.
